# The York County Hospital Inquiry

**Published:** 1913-06-07

**Authors:** 


### The Hospital

The Workers' Newspaper of Administrative Medicine and Institutional Life, Administration, National Insurance and Health.

No. 1406, Vol. LIV SATURDAY, JUNE 7, 1913. PRICE ONE PENNY.

### THE YORK COUNTY HOSPITAL INQUIRY.

In another part of our present issue will be found the full report of Sir Cooper Perry and Mr. Basil Watson, his legal assessor, who were appointed to conduct an inquiry into the charges made against this hospital by Mr. J. G. Macqueen, M.B. Edin., the late house surgeon, and Mr. W. Moir Shepherd, M.B. Edin., the late house physician to the York County Hospital, which we published in our issue of March 15, pages 645 *et seq.*, and to furnish the Committee of this hospital with a report on the result of their investigations. The verbatim report of the whole of the evidence taken before the Commission was published in The Hospital of April 26, a remarkable instance of what modern journalism can accomplish, in view of the fact mentioned by Sir Cooper Perry that he did not receive the complete transcript of the shorthand notes of the evidence, about 76,000 words, until May 5 following. The report is a very able document and testifies to the character, knowledge, and impartiality with which the inquiry has been conducted and the report drawn. The Commissioners point out that it is no part of their reference to make suggestions for the avoidance in future of such regrettable incidents as the present inquiry has disclosed. The full responsibility for action and reform therefore rests with the governors of the York County Hospital, a fact which we shall keep fully in mind in dealing with the report at this stage of the matter. That the inquiry should have disclosed the many and regrettable incidents referred to makes it clear that if the York County Hospital is to attain to the high standard of efficiency which characterises the modern up-to-date voluntary hospitals of the present day prompt action must be taken by the governors, and radical changes will have to be introduced into the administration of the institution with as little delay as possible.

It may be well to recall the circumstances which preceded the inquiry and the causes which led up to it. In The Hospital of January 27, 1912, page 433, we published a report on the York County Hospital by Sir Henry Burdett of the condition in which he found it at the date of his inspection. After comparing the hospital at that date with what it was sixteen years before, he reported: "The impression we gained from a thorough inspection of this hospital was that the internal administration requires modernising from top to bottom; there seems to be too little direct touch from the centre with the actual duties of each sister, nurse, and probationer. Indeed, we gathered that the efficiency of the management would be materially increased by the introduction of a controlling hand backed by up-to-date knowledge; for, although there is no intentional inefficiency, slackness and the absence of modern methods in several directions are apparent throughout the hospital." Those who have read the evidence before the Commissioners and who study their report must be impressed with the feeling that the foregoing was an accurate statement of the internal condition of this institution as now revealed. This report was published some months before the appointment of Messrs. Macqueen and Shepherd to the posts of resident medical officers, and they, in fact, had no knowledge of Sir Henry's report until shortly before they terminated their connection with the institution. It was a courageous and public-spirited course for the two residents to take to make the report which we published, upon which the inquiry was based. It was a remarkable document as it stood, and before we gave it publicity we were careful to confer with Professor Littlejohn, Dean of the University of Edinburgh, who reported that the authors were men whose statements as to facts we need have no hesitation in accepting; that they were both young men and had not had much experience, so that, although the Professor would probably not be prepared to accept all their views in regard to hospital management and administration, he would accept their statement on the general facts. Whatever else the report of the Commissioners may be held to prove, it certainly upholds the correctness of the testimonial given to the residents by Professor Littlejohn. We congratulate Messrs. Macqueen and Shepherd upon the courage they have displayed and the disinterested course they took, which cannot fail to have widespread results for good, for it must materially strengthen the position of junior residents, who often have to face regrettable difficulties, in the smaller hospitals especially. The publicity which has been given to the matter cannot fail to add to the difficulties the authorities at York may experience in procuring applicants for the residential posts, unless or until they put their house in order and take the public, the medical authorities, and the Universities and colleges into their confidence.

We do not propose to-day to express any view as to particular findings of the Commissioners. Any views we may hold must clearly be deferred until the governors of the York County Hospital have had full opportunity to consider the report in all its bearings and to deal with it on its merits, having regard to the future interests and reputation of their hospital. We may, however, properly state the result so far as the twenty-six charges made by the late residents against the hospital are concerned, which the Commissioners have investigated and which they deal with in the order in which they appeared in our columns on March 15 last. Out of twenty-five charges the Commissioners find that the following thirteen charges were justified or in the main admitted to be correct, or that the residents were right in their suspicions or dissatisfaction: The alleged ill-treatment of a child; mis-charting in the children's ward; alleged inadequacy of the nursing staff; complaints against the matron; children tied down in bed; a patient removed to the mortuary (with the exception that the Commissioners are of opinion that the residents left the patient in the mortuary after their first discovery that he was still alive and did not actually remove him until the assistant matron interrupted their luncheon); failure to report a burn on a child; delay in taking cases to the operation theatre; laxity in carrying out orders; failure to chart pulse and respiration; negligence in reporting deaths; absence of the day staff from the wards; retirement of residents; question of testimonials. In regard to two others, the treatment of sick nurses, the specific instances of alleged neglect were not sustained, but the Commissioners find it is clear there is much need for a nurses' sick room so placed that it can be conveniently worked in connection with the ward, and they recommend the provision of a sick room for nurses. In reference to the alleged unhappiness of the nursing staff, the Commissioners took no evidence, and they do not know what instances the residents had in mind. The following two charges are not denied: lack of ward etiquette; misconduct of a sister. In the following seven cases the charges are not substantiated or confirmed, or lack substance:—Treatment of surgical shock; washing of a patient suffering from shock; the slipper case; alleged inefficiency of a "special" nurse; alleged incompetence of a ward nurse; food and personal laundry; the condition of the residents' quarters. It would thus appear that of the twenty-six* charges fourteen stand; two partially stand and are found to have some substance; two are not denied; and seven are dismissed.

The Commissioners make a strong point, in their report on the charge of the alleged ill-treatment of a child, that the hospital authorities by the evidence they produced endeavoured to show that this case had importance from its general bearing on the whole attitude of Messrs. Macqueen and Shepherd and the charges they had made against the hospital, but the Commissioners take a contrary view, and have formed the opinion that the residents did actually see marks upon the child which were the results of the ill-treatment he received from the sister. They report as to this allegation that the result of the investigation undertaken by the Medical Board justified the residents in making this complaint.

It remains to be seen what course the authorities and governors of the York County Hospital will now take. We regret to see that the *Yorkshire Herald*, which appears to have voiced the view of the hospital authorities throughout this matter, maintains, despite the findings of the Commissioners, that "the more serious allegations are declared to be unsubstantiated," and "generally speaking, the hospital staff is exonerated by the report from any serious failure of duty hurtful to the patient." The view taken is that the dismissal of a sister and the action taken by the authorities after the complaints made by the late residents have removed the causes of the evil and *mirabile dictu* "the most shocking part of the charges, that relating to the case of a man said to have been taken alive to the mortuary, is disposed of in a rather dramatic manner for the ex-residents." The *Yorkshire Herald* finally holds that "very few public hospitals could undergo such an investigation and stand in so creditable a position." This last statement shows an utter ignorance of the high standard of efficiency maintained in the majority of our voluntary hospitals to-day. We can only hope that the utterances we have quoted in no sense represent the opinions of the governors and people of York or of the medical staff of the York County Hospital. It has been well said in regard to voluntary hospitals in this country that the citizens deserve the efficient, or inefficient, hospital which their city may contain. We cannot credit that the report of the Commissioners will be accepted in the spirit indicated by the quotations we have just given. If it is, such a decision cannot prove in practice to promote the interests of the sick and suffering residents of the city of York who may require hospital care when struck down by accident or overtaken by disease.

* There are practically twenty-five charges, for the Report amalgamates charges 3 and 9.

