# Errata

**Published:** 1913-06-14

**Authors:** 


In the leading article "The York County Hospital Inquiry," published on pages 293 and 294 of last week's issue, in the second paragraph, first column, p. 294, line 23, the word "fourteen" should be substituted for "thirteen." In the footnote to the same article, by a printer's error, "9" was substituted for "19," the charge referred to.

